# *Vibrio alginolyticus* Survives From Ofloxacin Stress by Metabolic Adjustment

**DOI:** 10.3389/fmicb.2022.818923

**Published:** 2022-03-16

**Authors:** Yue Yin, Yuanpan Yin, Hao Yang, Zhuanggui Chen, Jun Zheng, Bo Peng

**Affiliations:** ^1^State Key Laboratory of Biocontrol, Guangdong Key Laboratory of Pharmaceutical Functional Genes, School of Life Sciences, Southern Marine Science and Engineering Guangdong Laboratory (Zhuhai), Higher Education Mega Center, Sun Yat-sen University, Guangzhou, China; ^2^Laboratory for Marine Biology and Biotechnology, Qingdao National Laboratory for Marine Science and Technology, Qingdao, China; ^3^Department of Pediatrics, The Third Affiliated Hospital of Sun Yat-sen University, Guangzhou, China; ^4^Faculty of Health Sciences, University of Macau, Taipa, Macau SAR, China

**Keywords:** *Vibrio alginolyticus*, ofloxacin, metabolomics, pyruvate cycle, fatty acid synthesis

## Abstract

Antibiotic-resistant *Vibrio alginolyticus* becomes a worldwide challenge threatening both human health and food safety. The approach in managing such infection is largely absent, despite the fact that the mechanisms of antibiotic resistance have been extensively investigated. Metabolic modulation has been documented to be a novel approach in improving antibiotic efficacy. In this study, we characterize the metabolic signature of *V. alginolyticus* exposed to 0.3 or 0.5 μg/ml of ofloxacin (OFX). By profiling the metabolome, we find that bacteria treated by the two different concentrations of OFX generate different metabolic signatures. While a part of these metabolites was shared by both groups, the other metabolites represent their own signatures. The pathway enrichment analysis demonstrates that the pyruvate cycle is disrupted in the bacteria treated by the 0.3 μg/ml OFX as compared to those by the 0.5 μg/ml. Importantly, the disruption of pyruvate cycle confers the capability of bacteria to survive under 0.5 μg/ml of antibiotic stress. Further analysis identifies that the fatty acid biosynthesis is elevated in bacteria treated by 0.3 μg/ml OFX, and inhibition on fatty acid completely prevents the bacteria from survival even under such dose of antibiotic stress. Our study suggests that bacteria adapt to antibiotic stress by modulating the metabolic flux for survival, which could be targeted to increase antibiotic efficacy.

## Introduction

*Vibrio* spp., a group of Gram-negative bacteria, are prevalent in freshwater, estuarine, and marine environments (Baker-Austin et al., 2017). These bacteria are highly adaptable to different environments and tolerant to various adverse conditions (Baker-Austin et al., 2018). They represent one of the dominant species in seawater. Some of the Vibrios, such as *Vibrio alginolyticus*, *V. parahemolyticus*, and *V. vulnificus*, are pathogenic to both aquatic animals and human beings (Ina-Salwany et al., 2019). *V. alginolyticus*, for example, infects a wide range of economical aquatic animals including shrimp, fish, and shellfish. Infected fish are commonly associated with red spots on the ventral and lateral areas, swollen and dark skin lesions, and the release of blood exudate, known as vibriosis (Novoa et al., 1992). In addition, *V. alginolyticus* also causes bacteremia in fish that are exemplified as exophthalmos, ascites, and inflated spleen (Gong et al., 2020). Moreover, *V. alginolyticus* can rapidly kill the fish host before any symptom can be identified. *V. alginolyticus* can cause ear and wound infection in humans after being exposed to contaminated water (Jones et al., 2013). Therefore, *V. alginolyticus* is a harmful species that causes huge economic loss in aquaculture and presents a potential threat to human health.

Antibiotics is the primary choice to treat *V. alginolyticus* infection. Quinolone is a representative group of antibiotics that have been widely used in aquaculture in the past decades, owning to its high killing efficacy to different species of the genus *Vibrio* (Backhaus et al., 2000). The mechanism underlying the quinolone-mediated killing of bacteria is that it works as an inhibitor to the DNA gyrase, which belongs to the type II topoisomerases that are mainly involved in the topological transition of DNA (Reece and Maxwell, 1991). A quinolone antibiotic can result in the inhibition on DNA replication, repairment, recombination, and transposition, and thus disrupt RNA and protein biosynthesis (Paton and Reeves, 1988; Just, 1993), leading to a bactericidal effect (Baquero and Levin, 2021). However, the overuse of antibiotics including quinolones in aquaculture generates antibiotic-resistant bacteria. Clinically, multidrug-resistant *Vibrios* were frequently isolated from a variety of seafood in many countries, including China, Korea, and the United States (Zhao et al., 2011; Gong et al., 2020). Quinolone-associated resistance mainly results from the genetic mutations on the quinolone resistance-determining region (QRDR) of the gyrase gene, the decrease in membrane permeability, the increase in efflux pump, or plasmid-mediated resistance (Okuda et al., 1999; Otsuka et al., 2005; Xia et al., 2013; Hamed et al., 2018; Zhou et al., 2019).

Antibiotic resistance is strongly linked to bacterial metabolism (Stokes et al., 2019). Lab-evolved and clinic isolated kanamycin-resistant bacterial strains have different metabolomes to kanamycin-sensitive strain (Peng et al., 2015), even though the resistant mechanism was undefined. The identification of glucose, alanine, fructose, and glutamate as decreased metabolites in abundance in antibiotic-resistant bacteria indicates that metabolites could be potential regulators of antibiotic resistance. The exogenous supplementation of these substances could reverse antibiotic resistance through promoting antibiotic uptake; thus, a high concentration of antibiotics could be fluxed into the bacteria and exceed the threshold that bacteria can tolerate. By this way, the kanamycin-resistant bacteria were resensitized to kanamycin (Peng et al., 2015; Su et al., 2015, 2018; Cheng et al., 2019; Zhang et al., 2020). Similarly, a recent report identified that glutamine and inosine were decreased in abundance in multidrug-resistant bacteria. The exogenous supplementation of glutamine and inosine potentially augmented the antibiotic efficacy, especially for β-lactams. These two metabolites increased antibiotic influx into the bacteria that overcome the effect by an efflux pump and hydrolysis by β-lactams (Zhao et al., 2021). In addition, metabolites, such as glucose and alanine, not only promote antibiotic killing but also promote the generation of reactive oxygen species, which is downregulated in antibiotic-resistant *V. alginolyticus*; the generation of reactive oxygen species (ROS) promotes killing (Ye et al., 2018; Zhang et al., 2019, 2020). Moreover, the deletion of sodium-pumping complex (NAQ) complex confers antibiotic resistance in *V. alginolyticus*, but it can also be reversed through metabolite supplementation (Jiang et al., 2020). Metabolite-enhanced antibiotic killing is applicable not only to antibiotic resistance but also to antibiotic tolerance (Vega et al., 2012). Therefore, the identification of metabolites able to significantly reverse antibiotic resistance is urgently needed. In this study, we monitored the global metabolic responses of *V. alginolyticus* to ofloxacin (OFX) and found that OFX-mediated killing required the activation of pyruvate cycle and the inhibition of fatty acid synthesis.

## Materials and Methods

### Bacterial Strains and Culture Conditions

*V. alginolyticus* ATCC 33787 was grown at 30°C in Luria–Bertani (LB) broth (pH 7.0) supplemented with 3% sodium chloride. The overnight culture was diluted 1:100 (v/v) in fresh LB broth (supplemented with 0, 0.3, or 0.5 μg/ml OFX) at 30°C for 2 h. The bacteria were harvested by centrifugation at 8,000 rpm for 3 min, and the cells were washed and resuspended in sterile saline and adjusted to an optical density (OD600) of 1.0. To build a growth curve, the bacterial growth was examined by measuring the OD600 of the culture at the indicated time. To study the effect of antibiotics, inhibitors of pyruvate cycle, or fatty acid biosynthesis on bacterial growth, the bacterial growth was examined by measuring the OD600 of the bacterial cultures at 10 h in medium with OFX, malonate, or triclosan (Sangon Biotech, Shanghai, China). At least three biologic replicates were performed.

### Measurement of the Minimum Inhibitory Concentration

The Minimum Inhibitory Concentration (MIC) was determined by antimicrobial susceptibility testing following the previous description. In brief, an overnight bacterial culture grown in 3% NaCl was diluted 1:100 (v/v) in fresh LB medium and cultured at 30°C, to an OD600 of 0.5. Bacterial cells equivalent to 10^5^ colony-forming unit (CFU) were dispensed into each well of a 96-well microtiter polystyrene tray that contained a series of 2-fold dilutions of antibiotic. Following 16 h incubation at 30°C, the bacterial growth was examined by measuring the OD600 of the culture. The MIC was defined as the lowest antibiotic concentration that inhibited visible growth. Three biological repeats were carried out.

### Metabolomics Analysis

Bacterial sample preparation was carried out as previously described (Du et al., 2017). The bacteria were harvested by centrifugation at 8,000 rpm for 3 min, and the cells were washed and resuspended in sterile saline and adjusted to 1.0 at OD600. About 10 ml bacteria with OD600 = 1.0 were quenched with cold methanol and sonicated for 10 min at 200 W. Samples were centrifuged with12,000 rpm at 4°C for 10 min. A supernatant, containing 1 mg/ml ribitol (Sigma-Aldrich, St. Louis, MO, United States) as internal analytical standard, was transferred into a new tube and dried by a vacuum centrifugation device (LABCONCO). The dried extracts were then incubated with 80 μl methoxyamine hydrochloride (20 mg/ml, Sigma-Aldrich, St. Louis, MO, United States) in pyridine (Sigma-Aldrich, St. Louis, MO, United States) for 180 min at 37°C, and the derivatization was done with an identical volume of N-methyl-N-(trimethylsilyl) trifluoroacetamide (Sigma-Aldrich, St. Louis, MO, United States) for another 45 min. Samples were centrifuged at 12,000 rpm for 15 min, and the supernatant was transferred into new tubes. Gas chromatography–mass spectrometry (GC-MS) analysis was performed with an Agilent GC-MS instrument. By using spectral matching and retention time (RT) indexes from the National Institute of Standards and Technology (NIST) library of the NIST MS search 2.0, metabolites were identified. Metabolites were determined after removing known artificial peaks and merging the identical compounds. The resulting data matrix was normalized by the concentrations of added internal standards and the total intensity. Normalized peak intensities formed a single matrix with Rt-m/z pairs (retention time–mass charge ratio pairs) for each file in the data set. The matrix can be used for further analysis. According to a reference distribution, Z score analysis was used to scale each metabolite. Statistical difference was obtained by the Kruskal–Wallis test and Mann–Whitney test using solutions statistical package for the social sciences (SPSS) 13.0. Values with *p* <0.01 were considered as significant. Hierarchical clustering was completed in the R platform with the package gplots1 using the distance matrix. Multivariate statistical analysis included principal component analysis (PCA) and orthogonal partial least square discriminant analysis (OPLS-DA) implemented with SIMCA 12.0.1 (Umetrics, Umeå, Sweden). Control scaling was selected prior to fitting. All variables were mean centered and scaled to the Pareto variance of each variable. PCA was used to reduce the high dimension of the data set. We analyzed the differential metabolites to their respective biochemical pathways as outlined in the MetaboAnalyst 3.0^1^ (Bala et al., 2021). Pathways were enriched by *p*-value < 0.05.

### Real-Time Quantitative PCR

Quantitative real-time PCR (qRT-PCR) was carried out as previously described (Li et al., 2016). About 1 ml of bacterial culture with OD600 = 1.0 was harvested. The total RNA of each sample was isolated with Trizol (Invitrogen, Carlsbad, CA, United States). Reverse transcription- PCR was conducted by using an EvoM-MLV RT kit with gDNA clean for quantitative PCR (qPCR) (AG11705; Accurate Biotechnology, Guangdong, China) with 1 mg of total RNA according to the manufacturer’s instructions. qRT-PCR was performed in 384-well plates with a total volume of 10 μl containing 5 μl 2 × SYBR green premix pro Taq HS qPCR kit (AG11701; Accurate Biotechnology, Guangdong, China), 2.6 μl H_2_O, 2 μl cDNA template, and 0.2 μl each of forward and reverse primers (10 mM). The primers are listed in Supplementary Table 1. All samples were tested in biological triplicate and run on the LightCycler 480 system (Roche, Mannheim, Germany) according to the manufacturer’s instructions, and four independent samples were assayed for both the control group and the test group. The cycling parameters were 95°C for 30 s to activate the polymerase; 40 cycles of 95°C for 10 s; and 56°C for 30 s. Fluorescence measurements were performed at 72°C for 1 s during each cycle. Cycling was terminated at 95°C with a calefactive velocity of 5°C/s to obtain a melting curve. Data are shown as the relative mRNA expression compared with control with the endogenous reference 16S rRNA gene.

### Measurement of the Activity of Enzymes in the Pyruvate Cycle

The determination of pyruvate dehydrogenase (PDH), alpha-ketoglutarate dehydrogenase (KGDH), succinate dehydrogenase (SDH), and malate dehydrogenase (MDH) activity was carried out as previously described (Cheng et al., 2017). The activity of phosphoenolpyruvate carboxykinase (PEPCK) enzymatic activity was performed through the PEPCK Assay Kit according to the manufacturer’s manual (Suzhou Comin Biotechnology, Suzhou, China). *V. alginolyticus* cultured in medium with OFX was collected and washed three times with sterile saline. The bacterial cells were suspended in × 1 PBS (pH 7.0) to OD600 = 1.0. Samples of 30 ml were collected by centrifugation at 8,000 rpm for 5 min. Pellets were resuspended in phosphate-buffered saline (PBS), and the cells were lysed by sonication for 10 min (200 W total power with 35% output, 2 s pulse, 3 s pause over ice). The solution was then centrifuged with 12,000 rpm at 4°C for 10 min to remove insoluble materials. The protein concentration of the supernatant was quantified by the BCA protein concentration determination kit (Beyotime, P0009). Then, 200 μg proteins were used for the determination of enzyme activity.

### Nicotinamide Adenine Dinucleotide – Hydrogen Measurement

Nicotinamide adenine dinucleotide – hydrogen measurement (NADH) measurement was carried out as described previously (Su et al., 2018). *V. alginolyticus* cultured in the presence of different concentrations of OFX were collected, washed, and resuspended in NADH extraction buffer. Then, extracts were heated at 60°C for 5 min. After the NAD extraction buffer was added, the extracts were vortexed and centrifuged at 12,000 rpm for 5 min. Supernatant was then collected for the measurement of NADH with the EnzyChrom NAD/NADH Assay Kit, following the user manual (BioAssay Systems).

### Measurement of Adenosine Triphosphate

The adenosine triphosphate (ATP) level in the cells was determined by a BacTiter-Glo™ Microbial Cell Viability Assay (Cat. G8231; Promega, Madison, WI, United States) as previously described (Cheng et al., 2019). In brief, bacterial cells were harvested after being treated by centrifugation at 8,000 rpm for 3 min and were then washed twice with sterile saline. Cells were resuspended with saline solution and adjusted to OD600 ≈0.2. Then, 50 μl samples were added to the well in a 96-well plate and mixed with an equal volume of the kit solution. The absorbance of the solution was measured using VICTOR X5 (PerkinElmer, Turku, Finland) according to the manufacturer’s instructions. The concentration of ATP was calculated according to the standard curve of ATP.

## Results

### Responses of *Vibrio alginolyticus* to Different Concentrations of Ofloxacin

**FIGURE 1:**
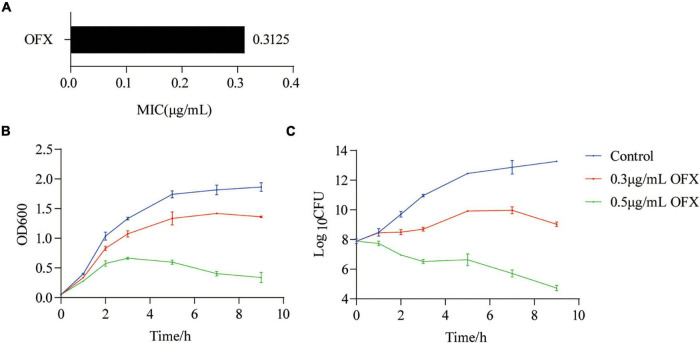
Killing curves of ofloxacin on V. *alginolyticus*. **(A)** MIC of *V. alginolyticus.* **(B)** Growth curve of *V. alginolyticus* in medium with the indicated concentrations of OFX. **(C)** CFU of **(A)**. Each data point represents means ± standard errors of the means (SEM) from three replicates.

To determine the optimal time point to analyze the metabolic response of bacteria to ofloxacin stress, *V. alginolyticus* was exposed to either 0.3 or 0.5 μg/ml OFX, corresponding to 1 or 1.6 × MIC of the parental strain, respectively (Figure 1A). In a time-dependent killing experiment, the number of viable bacteria was consistent at different time points during the whole course of antibiotic treatment by 0.3 μg/ml. However, the OD600 was increased at the first 7 h and began to decline slightly afterward (Figures 1B,C). In contrast, the number of viable bacteria dropped significantly when the bacteria were exposed to 0.5 μg/ml OFX, even though the OD600 was increased at the first 3 h (Figures 1B,C). The discrepancy of optical density (OD) value and viable counts might be due to the effect of cell filamentation at low antibiotic concentration where a filament with many cells produces a single colony. These data suggest that bacteria mounted a differential response to antibiotic stress as early as 1 h post-treatment of OFX. These differences became more significant at 2 h post-treatment of OFX that either continued to grow (control) or kept at a constant level of growth (0.3 μg/ml), or decreased the growth (0.5 μg/ml). So, 2 h antibiotic stress gives a good time point for the following analysis when the bacteria begin to die but OD is similar among the three groups. More importantly, the bacteria being treated with 0.3 μg/ml had a constant viability throughout the time-course experiment, suggesting that a survival mechanism might be present as compared to the bacteria being treated with 0.5 μg/ml.

### Metabolomic Profiles of *Vibrio alginolyticus* to Ofloxacin Exposure

**FIGURE 2:**
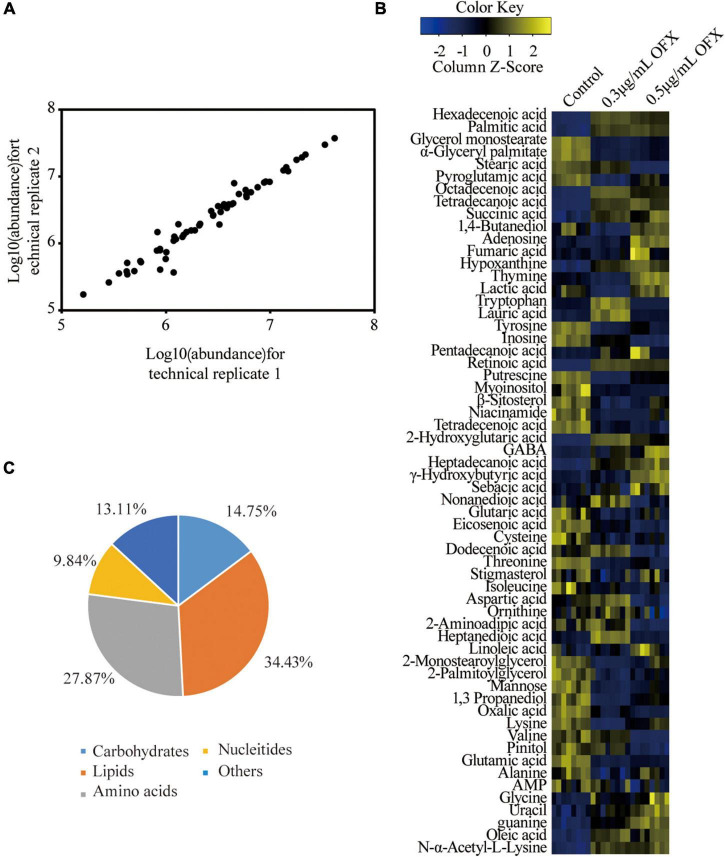
Metabolomic profiles of V. *alginolyticus* in response to OFX. **(A)** Heat map of identified metabolites. Scale is shown at bottom, where blue to yellow represents low-to-high abundance. **(B)** Reproducibility of metabolomic profiling. **(C)** Functional categories of the identified metabolites.

Bacteria culture was collected 2 h post-treatment of OFX for gas chromatography–mass spectrometry (GC-MS)-based metabolomic profiling for all of the three groups. Four biological replicates were included in each group, and each biological replicate had two technique replicates, altogether generating a total of 24 data points. The reproducibility of the data was demonstrated by Pearson correlation coefficients, showing low variation between the replicates (the relative standard deviation: 1.47% ± 0.03%) (Figure 2A). These results suggested that the differential abundance of the metabolites observed was attributed to different treatment but not to technique issues. Therefore, a total of 229 peaks were aligned, and the metabolites were identified by searching against NIST. The internal standard, ribitol, and any other known solvents were excluded. Finally, 60 metabolites were identified from all the three samples (Figure 2B). The identified metabolites belong to the functional categories of carbohydrate (14.75%), amino acids (27.87%), lipids (34.43%), nucleotides (9.84%), and unknown metabolites (13.11%), respectively (Figure 2C). These data together suggest that *V. alginolyticus* mounts metabolic shifts under ofloxacin stress.

### Metabolomic Signature of *Vibrio alginilyticus* to Different Concentrations of Ofloxacin

**FIGURE 3:**
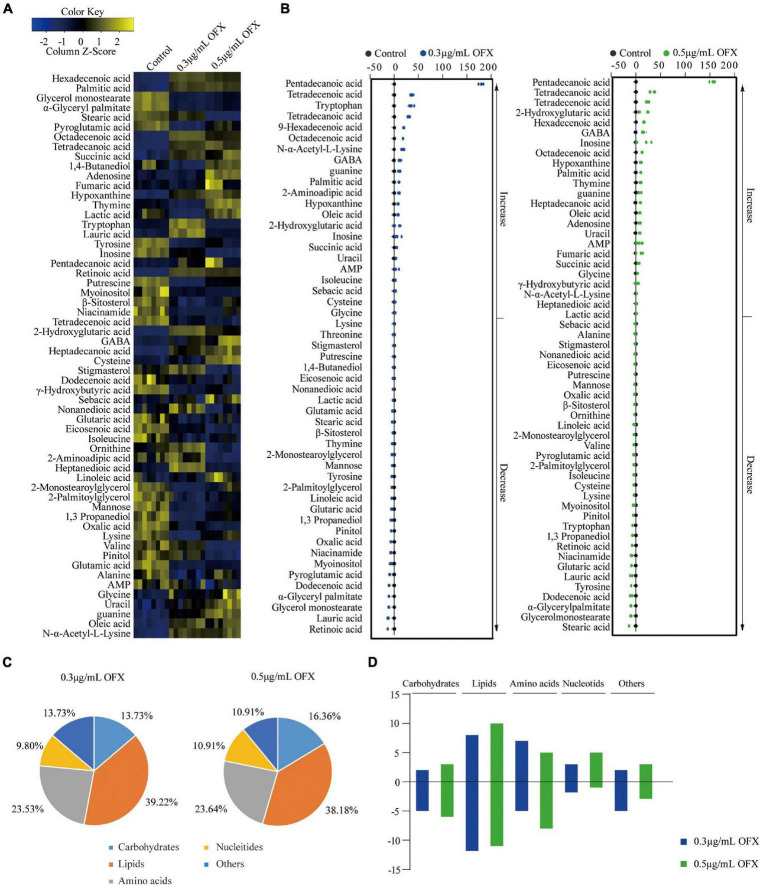
Differential abundance of metabolites in response to OFX. **(A)** Heat map showing relative abundance of metabolites (Wilcoxon *P* < 0.05) in control, 0.3 μg/ml OFX, and 0.5 μg/ml OFX groups. Scale is shown at bottom, where blue to yellow represents low-to-high abundance. **(B)** Z-score plot of differential metabolites based on control. Z-score varied between –6.63 and 89.50 for 0.3 μg/ml OFX to control, between –6.94 and 80.00 for 0.5 μg/ml OFX to control. Each point represents one metabolite in one technical repeat and is colored by sample types. **(C)** Percentage of differential abundance of metabolites in different functional categories. **(D)** The number of metabolites increased and decreased in different categories.

The different patterns in the growth of *V. alginotlyticus* exposed at different concentrations of OFX suggest that the bacteria treated by 0.3 μg/ml OFX may adapt differential metabolic responses to allow the bacteria to keep growing. Thus, a protective metabolic mechanism may exist to cope with OFX stress. To identify the metabolites with differential abundance, the Mann–Whitney test (Wilcoxon rank sum test) was adopted. A total of 51 and 55 differential metabolites were identified in the bacteria treated by 0.3 and 0.5 μg/ml OFX, corresponding to 0.5 and 0.69% false discovery rate, respectively, as compared to the control group (Figure 3A). The z-score, which demonstrates the distribution of the abundance of each metabolite around the mean, spanned from –6.63 to 89.50 in the 0.3 μg/ml group and from –6.94 to 80.00 in the 0.5 μg/ml. Accordingly, the abundance of 23 metabolites were increased and 28 metabolites were decreased in the 0.3 μg/ml group; 25 metabolites were increased and 32 metabolites were decreased in the 0.5 μg/ml group (Figure 3B). We analyzed the altered metabolites according to their functional categories and found that the differential metabolites under the category of lipids account for the most number of altered metabolites, followed by amino acids, then carbohydrates and other unknown metabolites, and nucleotides (Figure 3C). These data demonstrated that lipid metabolism and amino acid metabolism were significantly altered during the exposure. In addition, the number of altered metabolites between 0.3 and 0.5 μg/ml were different (Figure 3D). It is notable that the number of increased metabolites under the category of lipids in bacteria treated by 0.3 μg/ml OFX was more than the decreased metabolites. In contrast, a higher number of decreased metabolites under the category of lipids was observed in the bacteria treated by the 0.5 μg/ml OFX (Figure 3D).

Thus, bacteria mount a differential metabolic response to 0.3 and 0.5 μg/ml OFX treatment.

### Pathway Analysis of Differential Metabolites Upon Ofloxacin Treatment

**FIGURE 4:**
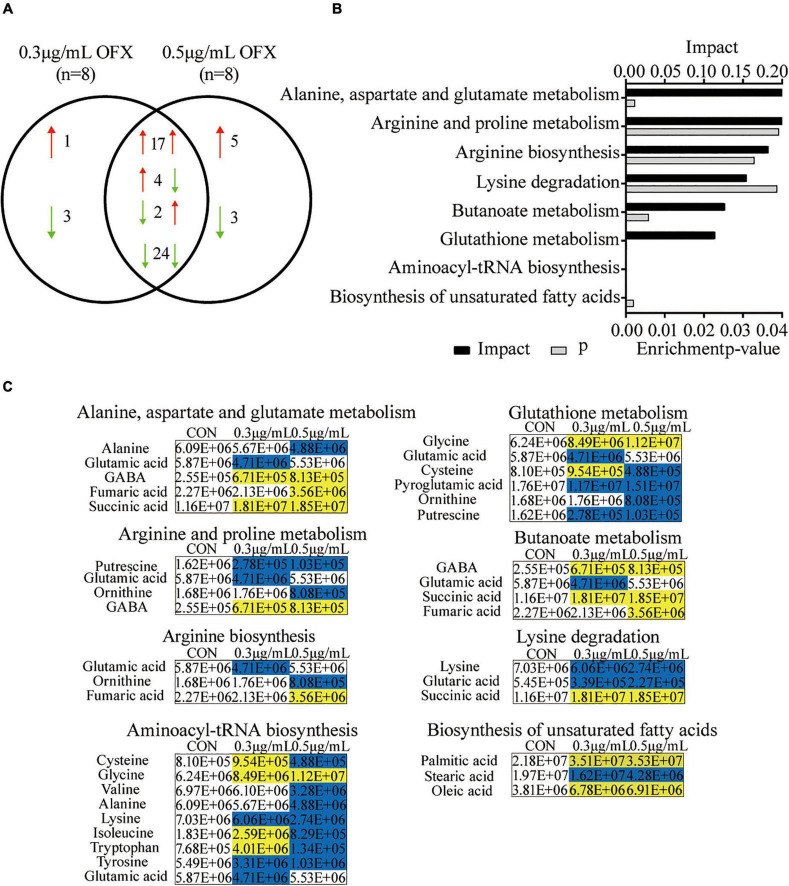
Pathway analysis of differential metabolites upon OFX treatment **(A)** Metabolites that were shared between the control, 0.3 μg/ml OFX, and 0.5 μg/ml OFX groups and their unique metabolites. **(B)** Enriched pathways by the shared metabolites. The metabolites of differential abundance were selected and analyzed in MetaboAnalyst to enrich pathways. Seven pathways that had significant difference (*P* < 0.05) were enriched and sorted by their weights (impact). **(C)** Differential metabolites in the enriched pathways.

Pathway enrichment analysis is of great importance to dissect the metabolic states influenced by antibiotics (Peng et al., 2015; Cheng et al., 2019; Zhao et al., 2021), despite the fact that the metabolites identified by GC-MS are biased toward organic acids. Thus, to get an idea about the difference between the 0.3 and 0.5 μg/ml groups, we further analyzed the differential metabolites. Among the 59 differential metabolites found in the bacteria treated by 0.3 and the 0.5 μg/ml OFX, 47 metabolites were shared by the two groups, where the abundance of 17 metabolites were increased; 24 metabolites were decreased in both of the groups. The abundance of four metabolites, tryptophan, isoleucine, sebacic acid, and L-cysteine, were increased in the 0.3 μg/ml group but decreased in the 0.5 μg/ml group. Another two metabolites, lactic acid and thymine, were decreased in the 0.3 μg/ml group but increased in the 0.5 μg/ml group (Figure 4A). Four metabolites, with one increased and three decreased metabolites, were specific to the 0.3 μg/ml group, and eight metabolites, with five increased and three decreased metabolites, were specific to the 0.5 μg/ml group (Figure 4A). The altered metabolites shared by both groups were analyzed by pathway analysis. A total of eight pathways were identified including alanine, aspartate, and glutamate metabolism; arginine and proline metabolism; arginine biosynthesis; lysine degradation; butanoate metabolism; glutathione metabolism; aminoacyl–tRNA biosynthesis; and the biosynthesis of unsaturated fatty acids (Figure 4B). It is interesting to note that the amino acids were significantly downregulated in the 0.5 μg/ml group but only a part of them was downregulated in the 0.3 μg/ml group, since the biosynthesis of amino acids was highly dependent on central carbon metabolism. This result suggests that central carbon metabolism was likely disrupted so that it fails to provide a sufficient substrate for macromolecule synthesis. In correlation with the decrease in the biosynthesis of amino acids, the intermediates metabolite (fumaric acid and succinic acid) of the pyruvate cycle, a novel metabolic cycle that provides energy to bacteria (Su et al., 2018; Zhang et al., 2020), was accumulated. This accumulation was much more significant in the 0.5 μg/ml group. The metabolite, γ-aminobutyric acid (GABA), that refills the succinate, was also accumulated in a dose-dependent manner (Figure 4C). However, the biosynthesis of amino acids was decreased in a dose-dependent manner. Since the altered metabolites of the pyruvate cycle in both of the 0.3 and 0.5 μg/ml groups showed the same trend of change, we postulate that pyruvate cycle might be critical for the survival under OFX stress.

### Disruption of Pyruvate Cycle Inhibits Ofloxacin-Induced Cell Death

**FIGURE 5:**
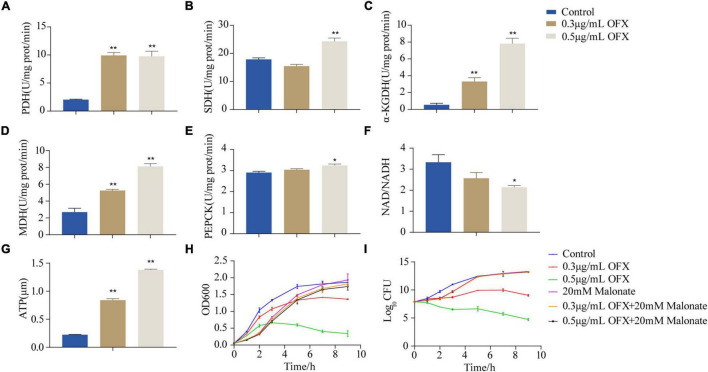
Disruption of pyruvate cycle inhibit OFX-induced cell death. **(A)** Activity of pyruvate dehydrogenase of pyruvate cycle upon OFX treatments. **(B)** Activity of succinate dehydrogenase of pyruvate cycle upon OFX treatments. **(C)** Activity of α-ketoglutarate dehydrogenase of pyruvate cycle upon OFX treatments. **(D)** Activity of malate dehydrogenase of pyruvate cycle upon OFX treatments. **(E)** Activity of phosphoenolpyruvate carboxykinase of pyruvate cycle upon OFX treatments. **(F)** NAD/NADH of *V. alginolyticus* in medium with the indicated concentrations of OFX. **(G)** ATP of *V. alginolyticus* in medium with the indicated concentrations of OFX. **(H)** Growth curve of *V. alginolyticus* in medium with the indicated concentrations of OFX or 20 mM malonate. **(I)** CFU of **(H)**. The statistical analyses were performed by one-way ANOVA, followed by LSD multiple comparison test, *P<0.05; **P<0.01. Error bars represent means ± SEM. Each experiment was performed in triplicate.

To confirm our hypothesis that pyruvate cycle was critical for OFX-mediated cell death, we quantify the enzymatic activity of pyruvate cycle in the bacteria treated by 0.3 and 0.5 μg/ml treatment, respectively, at 2 h post-treatment of OFX, the time point in collecting samples for GC-MS analysis (Figure 1B). Five enzymes including two pyruvate cycle-specific enzymes, PDH, and PEPCK and three enzymes in the canonical TCA cycle, succinate dehydrogenase (SDH), α-ketoglutarate dehydrogenase (α-KGDH), and MDH were included. Interestingly, the activity of PDH was increased in both of the groups and no significant difference was observed between the 0.3 and 0.5 μg/ml groups (Figure 5A). The SDH activity remained unchanged at the 0.3 μg/ml group but dramatically increased in the bacteria treated by 0.5 μg/ml OFX (Figure 5B), which was corroborated by the observation that fumarate was accumulated (Figure 4C). Interestingly, the α-KGDH was increased in a dose-dependent manner (Figure 5C), consistent with the gradual increase of the succinate level in the metabolomic data. The MDH activity increased 1.95-fold in the 0.3 μg/ml group, and 3.02-fold in the 0.5 μg/ml group (Figure 5D). The activity of PEPCK was unchanged at the 0.3 μg/ml group but increased in the bacteria treated by 0.5 μg/ml OFX (Figure 5E). As pyruvate cycle provides NADH, the aberrant pyruvate cycle suggests that it might be associated with the dysregulated redox potential that contributes to the ultimate cell death. Indeed, the ratio of NAD^+^/NADH was decreased in a dose-dependent manner (Figure 5F), indicating the preferred conversion of NAD^+^ to NADH. Consistently, the level of ATP content was also increased (Figure 5G). These data together suggest that the functional pyruvate cycle is associated with the promotion of OFX-mediated killing. Supportively, the block of the pyruvate cycle with malonate (20 mM), the inhibitor of succinate dehydrogenase, completely rescued the bacteria from the killing by either 0.3 or 0.5 μg/ml OFX, implying that pyruvate cycle was critical for OFX-mediated killing (Figures 5H,I).

### Identification of Crucial Metabolites Using Multivariate Data Analysis

**FIGURE 6:**
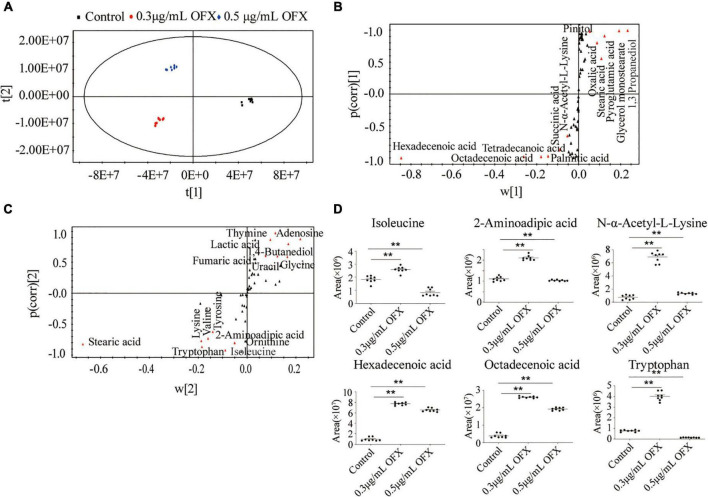
Identification of crucial metabolites using multivariate data analysis. **(A)** Principal-component analysis of control, 0.3 μg/ml OFX, and 0.5 μg/ml OFX groups according to the treatment set. Each dot represents the technological replicate analysis of samples in the plot. **(B)** S-plot generates from OPLS-DA. Predictive component *p*[1] and correlation *p*(corr)[1] differentiate control from 0.3 and 0.5 μg/ml OFX. **(C)** S-plot generates from OPLS-DA. Predictive component *p*[2] and correlation *p*(corr)[2] separate 0.3 μg/ml OFX from 0.5 μg/ml OFX. Triangles represent metabolites. Potential biomarkers are highlighted in red. **(D)** Abundance of crucial metabolites in control and 0.3 and 0.5 μg/ml OFX. Statistical analysis was performed with Mann–Whitney *U*-test. **P < 0.01.

To identify the metabolites that distinguish the 0.3 μg/ml group and 0.5 μg/ml group, OPLS-DA was adopted to recognize the sample patterns. The three groups, namely, the control, 0.3 μg/ml group, and 0.5 μg/ml group, were clearly separated from each other (Figure 6A). In detail, t[1] component separated the control group from the other two groups, whereas t[2] separated the 0.3 μg/ml group from the 0.5 μg/ml group (Figure 6A). S-plot, which allows the visualization both of covariance and correlation between the metabolites and the modeled class designation (Kalesinskas et al., 2018), was then used to identify the crucial biomarkers. As compared to the control group, the level of hexadecenoic acid, octadecenoic acid, palmitic acid, tetradecanoic acid, succinic acid and N-α-acetyl-L-lysine, stearic acid, oxalic acid, pyroglutamic acid, and 1, 3-propanediol were the most significant metabolites [variable important in projection (VIP) > 1, *p* < 0.05] in the 0.3 μg/ml group (Figure 6B). On the other hand, stearic acid, glycine, lysine, valine, tyrosine, 2-aminoadipic acid, tryptophan, isoleucine, ornithine, thymine, adenosine, lactic acid, 1, 4-butanediol, fumaric acid, and uracil were most significant metabolites in the 0.5 μg/ml group (VIP > 1, *p* < 0.05) (Figure 6C). Those crucial biomarkers were displayed as scatter plot in Supplementary Figure 1. We were particularly interested in the metabolite whose contents were increased in the 0.3 μg/ml group but decreased in the 0.5 μg/ml group since the bacteria in the 0.3 μg/ml group were surviving, whereas the bacteria in 0.5 μg/ml group died massively. By analyzing these metabolites, we may identify the metabolites that guarantee the cell survival. Such metabolites included isoleucine, 2- aminoadipic acid, N-α-acetyl-L-lysine, hexadecenoic acid, octadecenoic acid, and tryptophan (Figure 6D). The presence of two fatty acids implied that fatty acid synthesis might be important for cell survival.

### Fatty Acids Synthesis Is Important for Bacteria Survival Under Ofloxacin Stress

**FIGURE 7:**
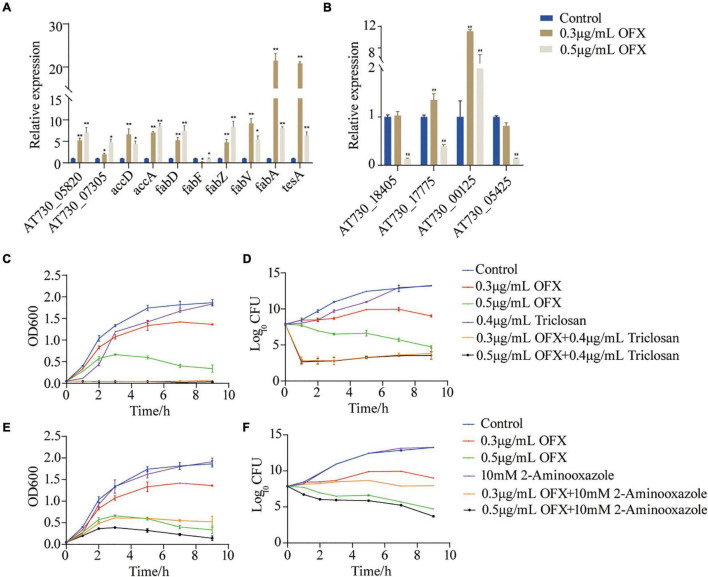
Fatty acid synthesis is a survival factor under OFX stress **(A)** Quantification of gene expression of fatty acid treated by indicated concentrations of OFX for 2 h. **(B)** Quantification of gene expression of unsaturated fatty acids treated by indicated concentrations of OFX for 2 h. **(C)** Growth curve of *V. alginolyticus* in medium with the indicated concentrations of OFX or 0.4 μg/ml triclosan. **(D)** CFU of **(C)**. **(E)** Growth curve of *V. alginolyticus* in medium with the indicated concentrations of OFX or 10 mM 2-aminooxazole. **(F)** CFU of **(E)**. The statistical analyses were performed with Mann–Whitney *U*-test unless otherwise indicated. *P<0.05; **P<0.01. Error bars represent means ± standard errors of the means (SEM). All of the experiments were repeated at least three times.

To identify whether fatty acid biosynthesis contribute to bacterial survival under OFX stress, the genes of the fatty acid synthesis was quantified (Figure 7A). The expression of all of the genes, except *fabF*, were increased upon OFX stress. The increase of *fabZ* expression was shown an OFX-dose-dependent manner, whereas *fabV*, *fabA*, and *tesA* increased most significantly in the 0.3 μg/ml group than those in the 0.5 μg/ml group (Figure 7A). To further confirm our observation, the expression of unsaturated fatty acid pathway, *AT730_18405*, *AT730_17775*, *AT730_00125*, and *AT730_05425*, was quantified by qRT-PCR. It was shown that the expression of *AT730_00125* were increased more significant in 0.3 μg/ml group than that in the 0.5 μg/ml group, and that *AT730_17775* was decreased in both groups (Figure 7B). To confirm the role of fatty acid synthesis in OFX stress, bacteria were treated by 0.3 or 0.5 μg/ml OFX in the presence triclosan and 2-aminooxazole, two inhibitors of fatty acid biosynthesis. Triclosan is a specific inhibitor to 3-oxoacyl-[acyl-carrier-protein] synthase II (Tkachenko et al., 2007). While 2-aminooxazole selectively targets acetyl-CoA carboxylase (Polyak et al., 2012). Triclosan alone could delay the growth of bacteria. The growth curve for bacteria treated by 0.3 or 0.5 μg/ml OFX were similar to that in Figure 1A. However, the presence of triclosan completely abolished the growth of both of the OFX-treated groups (Figures 7C,D). In addition, 2-aminooxazole alone has no impact on the growth of bacteria, but it decreases the survival upon OFX treatment (Figures 7E,F). The two inhibitors have different levels of synergistic effects, which may be due to their different targets. These data together suggest that fatty acid biosynthesis is important to cope with the OFX stress.

## Discussion

Tackling antibiotic resistance is an emerging topic throughout the world and requires urgent attention in both the policies and management of existing antibiotic-resistant bacteria (Tetteh et al., 2020). The development of a novel strategy in eliminating antibiotic-resistant bacteria has been researched for decades. The screening of novel compounds by high-throughput screening (Penchovsky and Stoilova, 2013) or artificial intelligence-based screening (Lau et al., 2021) or cultures from the unculturable bacteria (Lodhi et al., 2018) aids the finding of new chemicals that kill antibiotic-resistant bacteria without detectable resistance. These studies shed light on the innovation of new drugs that probably compensate for the currently exhausted antibiotic reservoir. However, the pipeline is still too long, and it takes around 10 years for an antibiotic to be successfully approved for clinical usage (Bush et al., 2011). Meanwhile, attempts on other methods, such as probiotic, bacteriophage-based approach, anti-virulence, and immunomodulatory molecules, have been proposed and studied for decades, but none is prompting in the clinic as well as not applicable in aquaculture. Therefore, resensitizing antibiotic-resistant bacteria to current available antibiotics becomes an urgent task that can overcome the shortage in the effective management approaches on combating antibiotic resistance.

Metabolism-based strategy offers a novel perspective to deal with antibiotic-resistant bacteria. Based on the hypothesis that metabolic state determines antibiotic efficacy, we and others have shown that metabolites, including alanine, glucose, fructose, glutamate, pyruvate, thymine, and serine, can reprogram the metabolomes of the antibiotic-resistant bacteria and resensitize the antibiotic-resistant bacteria to antibiotics *via* increasing antibiotic uptake, counteracting reactive oxygen species, generating nitric oxide, or interfering with the antibiotic binding to the target (Peng et al., 2015; Su et al., 2015; Perez-Morales and Bustamante, 2016; Ye et al., 2018; Zhang et al., 2020; Kuang et al., 2021). These data highlight that the metabolome of the bacteria can be remodeled through exogenous supplementation of metabolites, which is promising to be used to treat infectious diseases in clinic. Moreover, these metabolism-based methods were also applicable to fight serum-resistant bacteria. It can be used to boost host immunity to eliminate serum-resistant bacteria or directly enhance immunity including phagocytosis (Cheng et al., 2019; Yang et al., 2021a,b, c).

**FIGURE 8:**
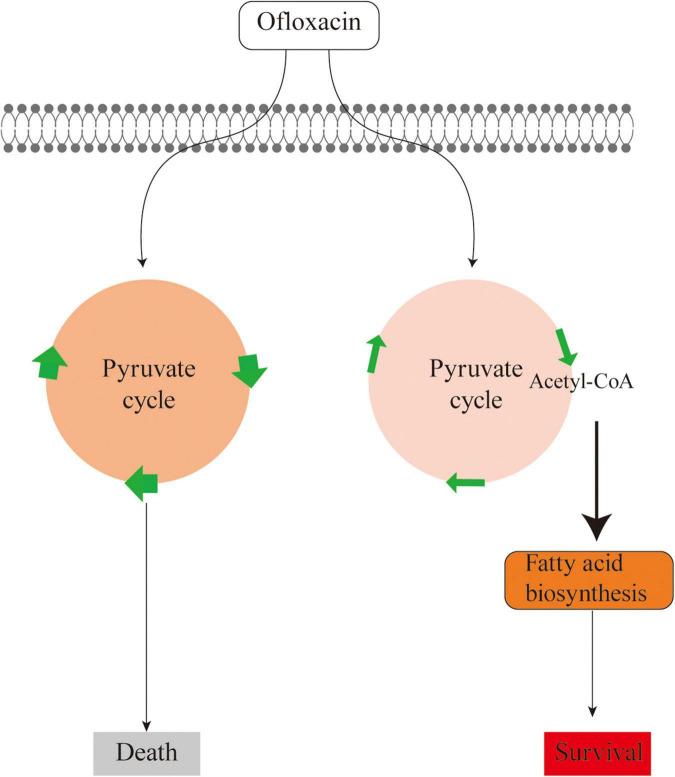
Sketch diagram describing a mechanism by which *V. alginolyticus* adapts to antibiotic stress.

Unlike the above-mentioned studies, this study explores the metabolic feature of *V. alginolyticus* upon the treatment by different concentrations of OFX. As compared to the antibiotic-resistant bacteria, the metabolic profile was much more dynamic during antibiotic stress. The expression of the pyruvate cycle genes or for fatty acid biosynthesis genes was quite different at various time-point post-treatments of OFX. One interesting observation here was that the bacteria treated by both concentrations of OFX had a higher level of pyruvate cycle and lower level of fatty acid synthesis. Despite small differences in concentrations, it is interesting to note that the bacteria mount such huge differences in the growth curve and enzymatic activity. This might be due to whether the intracellular concentration of antibiotics can exceed the threshold for the bacteria. Previous studies demonstrate that a minor difference of the intracellular concentration of antibiotics can tell apart the antibiotic-sensitive and antibiotic-resistant bacteria (Peng et al., 2015). The bacteria exposed to 0.5 μg/ml OFX might accumulate a higher level of antibiotics in the same period of time as to those exposed to 0.3 μg/ml OFX. Thus, it is highly possible that bacteria may require a decision-making to flux into the pyruvate cycle or to block the fatty acid synthesis, which needs to be further addressed (Figure 8). Moreover, another noticeable effect is that the bacteria at the MIC (0.3 μg/ml) can continue to grow, as shown in Figure 1. It is possible that the stage and doses of bacteria mattered. For the MIC experiments, 5 × 10^4^ CFU grown at logarithmic-phase bacteria was used, and kept in the incubator without shaking. But for the antibiotic stress experiment, overnight bacterial were diluted at 1:100 (v/v) in fresh medium to a final concentration as high as 10^7^ CFU, and more importantly, the diluted culture was kept with shaking. These differences may explain why the bacteria can still grow at MIC value.

Increased fatty acid biosynthesis has been studied in antibiotic-resistant bacteria. A recent report demonstrated that increased fatty acid biosynthesis can decrease the membrane fluidity and restrict the influx of antibiotics in norfloxacin-resistant bacteria (Su et al., 2021). Nevertheless, enhanced fatty acid biosynthesis was also reported in bacteria that are resistant to ceftazidime, levofloxacin, methicillin, and ciprofloxacin (Konai and Haldar, 2017; Cheng et al., 2018; Liu et al., 2019; Su et al., 2021). In addition, the inhibition of fatty acid synthesis can revoke the antibiotic sensitivity by two–fivefold (Su et al., 2021). It is interesting to observe that increased fatty acid biosynthesis promotes bacterial growth during antibiotic stress. The increase in fatty acid biosynthesis might be a strategy deployed by bacteria to maintain the membrane integrity as the membrane could be disrupted by antibiotic-induced ROS (Konai and Haldar, 2017; Ye et al., 2021). The altered transcription of fatty acid biosynthesis might be attributed to the antibiotic effect that reduces the transcription and translation of lipoproteins supporting cell membrane structure (Braun and Hantke, 2019). Therefore, the co-occurrence of increased fatty acid biosynthesis in both antibiotic stress and antibiotic-resistant bacteria imply that bacteria may coordinate fatty acid biosynthesis during the regime of antibiotic treatment. Thus, searching for effective inhibitors in inhibiting fatty acid biosynthesis may be a potential strategy in preventing the evolution of antibiotic-resistant bacteria during antibiotic treatment.

However, the limitation of this study is that we only chose two concentrations of antibiotics that may not be able to cover up other mechanisms employed by the bacteria to cope with antibiotics. It is certain to use a wider range of antibiotic concentrations, ranging from sublethal doses to lethal doses, to obtain a comprehensive view of the metabolic response upon antibiotic stress in the future study. In addition, we also cannot exclude the possibility that OFX triggered such metabolic changes. The viability of bacteria treated with 0.3 μg/ml OFX being constant may be due to the apoptotic-like bacterial death, which requires energy, and the following blockage of pyruvate cycle may deplenish the energy for cell death (Lewis, 2000). It is therefore necessary to identify the enzymes, for example, being targeted by OFX in addition to DNA gyrase.

In summary, our study demonstrates that *V. alginolyticus* mounts a differential metabolic response to different concentrations of OFX, which is associated with the consequences of treatment. By metabolomic profiling, pathway, and multivariate analysis, we found the metabolic flux to pyruvate cycle and fatty acid synthesis plays important roles. The inhibition of fatty acid biosynthesis was found to increase antibiotic efficacy. Thus, our study expands the understanding of metabolism-based approach to fight against antibiotic resistance.

## Data Availability Statement

The original contributions presented in the study are included in the article/Supplementary Material, further inquiries can be directed to the corresponding author/s.

## Author Contributions

YY, YPY and HY conducted the experiments. YY, YPY and ZGC performed data analysis. YY, JZ, and BP interpreted the data. BP wrote the manuscript, conceptualized, and designed the project. All authors reviewed the manuscript and acknowledged the contributions.

## Conflict of Interest

The authors declare that the research was conducted in the absence of any commercial or financial relationships that could be construed as a potential conflict of interest.

## Publisher’s Note

All claims expressed in this article are solely those of the authors and do not necessarily represent those of their affiliated organizations, or those of the publisher, the editors and the reviewers. Any product that may be evaluated in this article, or claim that may be made by its manufacturer, is not guaranteed or endorsed by the publisher.

## Footnotes

1
http://www.metaboanalyst.ca/
