# Supplementary material for: Vibrio alginolyticus Survives From Ofloxacin Stress by Metabolic Adjustment
Source: Front Microbiol. 2022 Mar 16;13:818923. doi: 10.3389/fmicb.2022.818923 (PMC8966707; doi:10.3389/fmicb.2022.818923)
Supplement: Supplementary file 1 [file Data_Sheet_1.docx]

**Supplementary Figure**


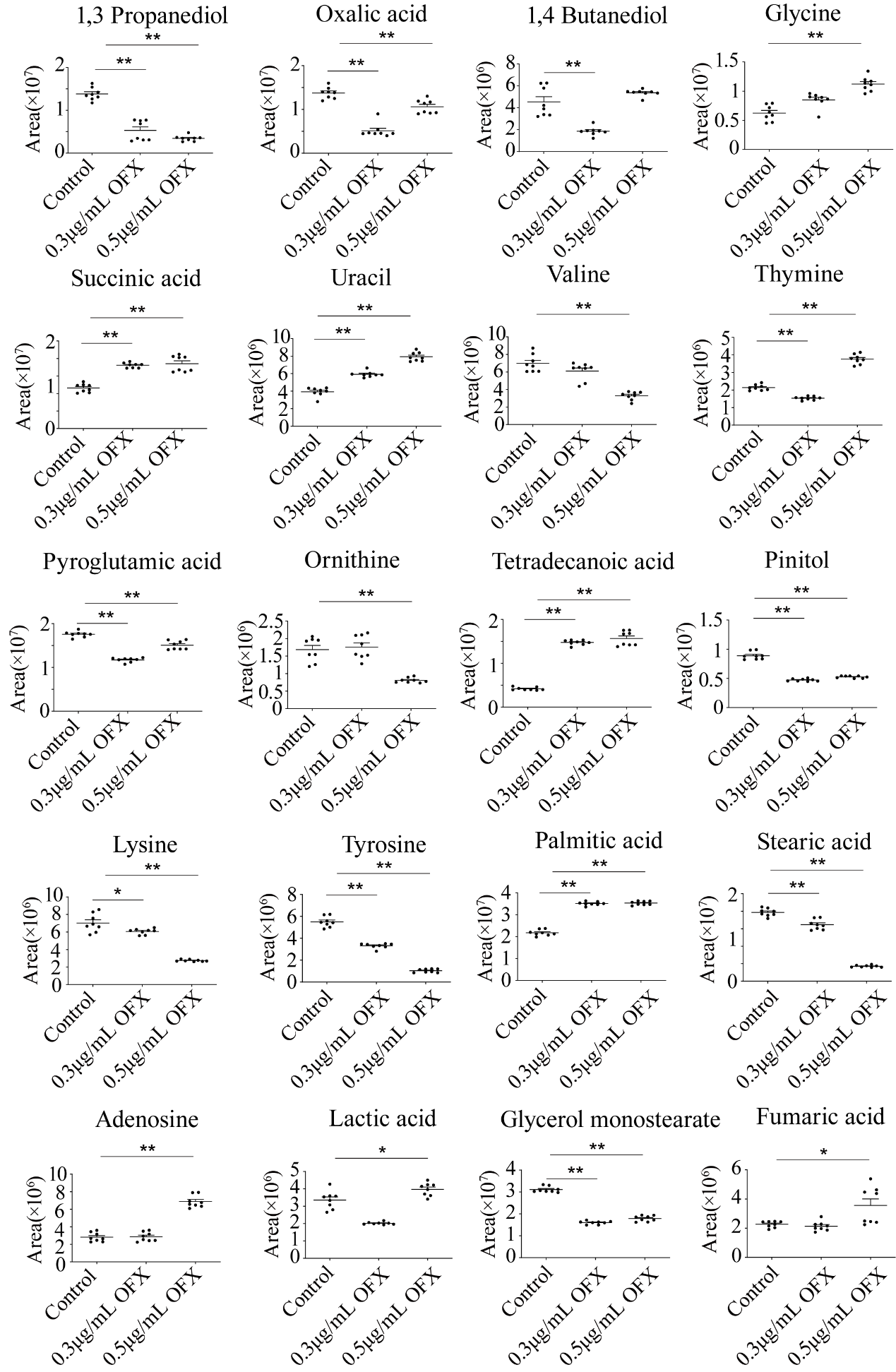


**Figure S1** Scatter plot of the differential metabolites in control, 0.3μg/mL OFX and 0.5μg/mL OFX group. Statistical analysis was performed with Mann-Whitney U test. *, P <0.05; **, P <0.01.

**Supplementary Table**

**Table 1** Primers used for qPCR of the *V. alginolyticus*

| Gene name | Former/Reverse Primer | Sequence |
| --- | --- | --- |
| *AT730_05820* | Former | 5’-ACTTAGCCGCTGCTTGTTCT-3’ |
|  | Reverse | 5’-GGTTGAACGGTCTGTCGGAT-3’ |
| *AT730_07305* | Former | 5’-GTTACCGGAGAGGCGTCAAA-3’ |
|  | Reverse | 5’-CGCATACCTCCGAGCATCAA-3’ |
| *accD* | Former | 5’-TGCTCAATGACCCGTCTTCC-3’ |
|  | Reverse | 5’-AACTTATGGTGGCGTCTCGG-3’ |
| *accA* | Former | 5’-AGTGACCTAGGTGCATGGGA-3’ |
|  | Reverse | 5’-CCCACAATCGCTTTGTCGTC-3’ |
| *fabD* | Former | 5’-TGCTTGCAGAACTAGGCGAA-3’ |
|  | Reverse | 5’-CCATTTTGAACAAGCGCCCA-3’ |
| *fabF* | Former | 5’-GAAAGGCCGAGTGATCCCAA-3’ |
|  | Reverse | 5’-CCAGCAACCATCACCGTTTG-3’ |
| *fabZ* | Former | 5’-GCGCGAAATTCCGTAAACCA-3’ |
|  | Reverse | 5’-ACTTCGCCGTCAACTTTTGC-3’ |
| *fabV* | Former | 5’-AACCTGCTGTACCTGGCTTC-3’ |
|  | Reverse | 5’-GCATCAAGTGGCTACGGTCT-3’ |
| *fabA* | Former | 5’-TTTCGCTGTTGGCAGGATCT-3’ |
|  | Reverse | 5’-TCTTGGCCTTGATGCGATGT-3’ |
| *tesA* | Former | 5’-TCCAAGGTTGTGCATCTGGT-3’ |
|  | Reverse | 5’-TGCAAATTCGGGTTCCACCT-3’ |
| *AT730_18405* | Former | 5’-GGCGCGCAAAATAGGATGAG-3’ |
|  | Reverse | 5’-TGGACTGGGTTGGGAACATC-3’ |
| *AT730_17775* | Former | 5’-GAGGTAACCGTGCTAAGCCA-3’ |
|  | Reverse | 5’-TGCCGATCGAAGAGAGTTGG-3’ |
| *AT730_00125* | Former | 5’-TGCCTCAAGTTTATGGCGGT-3’ |
|  | Reverse | 5’-CTGGATCGCCAGGGTACAAA-3’ |
| *AT730_05425* | Former | 5’-TAGCAGCATACGACATCGCC-3’ |
|  | Reverse | 5’-GTCTCAGCTCGACTTAGCGG-3’ |
| *16S* | Former | 5’-GTCGCTAACGTCAAATAATGCAGC-3’ |
|  | Reverse | 5’-TTGCACAATGGGCGCAAG-3’ |
